# Phenotypic variability between Social Dominance Ranks in laboratory mice

**DOI:** 10.1038/s41598-018-24624-4

**Published:** 2018-04-26

**Authors:** Justin A. Varholick, Jeremy D. Bailoo, Rupert Palme, Hanno Würbel

**Affiliations:** 1 Division of Animal Welfare, Veterinary Public Health Institute, University of Bern, Bern, Switzerland; 2 Department of Biomedical Sciences, University of Veterinary Medicine, Vienna, Austria

## Abstract

The laboratory mouse is the most prevalent animal used in experimental procedures in the biomedical and behavioural sciences. Yet, many scientists fail to consider the animals’ social context. Within a cage, mice may differ in their behaviour and physiology depending on their dominance relationships. Therefore, dominance relationships may be a confounding factor in animal experiments. The current study housed male and female C57BL/6ByJ mice in same-sex groups of 5 in standard laboratory conditions and investigated whether dominance hierarchies were present and stable across three weeks, and whether mice of different dominance ranks varied consistently in behaviour and physiology. We found that dominance ranks of most mice changed with time, but were most stable between the 2^nd^ and 3^rd^ week of testing. Phenotypic measures were also highly variable, and we found no relation between dominance rank and phenotype. Further, we found limited evidence that variation in measures of phenotype was associated with cage assignment for either males or females. Taken together, these findings do not lend support to the general assumption that individual variation among mice is larger between cages than within cages.

## Introduction

The laboratory mouse; *Mus musculus*, is the most widely used animal in biomedical and behavioural research. Despite their high prevalence of use, researchers rarely consider the social contexts of mice housed under laboratory conditions. Rather, it is generally assumed that mice housed within a cage are more phenotypically similar relative to mice housed between cages, because of their shared cage environment^1^. However, research on social dominance suggests that groups of mice within cages form dominance hierarchies, and that mice may vary phenotypically depending on their dominance rank^2–4^. Neglecting phenotypic variation within cages of mice, as a consequence of dominance rank, may potentially mask treatment effects^5,6^ and contribute to spurious experimental results^7,8^.

Dominance relationships and hierarchies were first described by Schjelderup-Ebbe^9^, and have since been identified in many social animals, including; fish, reptiles, birds, and mammals. Within a dominance relationship, one individual repeatedly and consistently yields to the other individual’s agonistic behaviour — thereby reducing the frequency of aggressive interactions^10^. The animal that consistently yields is considered subordinate to the other (dominant) animal. Once the dominance relationships between all dyads within a group (e.g., in a cage) are established, these relationships can be organized into a dominance hierarchy where each individual may have a unique dominance rank in relation to the other group members^11,12^. However, dominance hierarchies can vary in their organization. Classically, dominance hierarchies are organized in a linear or transitive structure where A is dominant over B and C, and B is dominant over C. In such a transitive structure A assumes the 1^st^ position (alpha), B assumes the 2^nd^ position (beta), and C assumes the 3^rd^ (gamma) or last position (omega). Groups may also form an intransitive structure where A is dominant over B, B is dominant over C, and C is dominant over A; or they may form a despotic structure where A is dominant over B and C, while B and C have no clear relationship. Thus, within intransitive or despotic structures dominance ranks may not be clearly defined. Groups composed of more than three individuals may include transitive, intransitive, and/or despotic sub-structures — making the overall structure of the dominance hierarchy complex^13^.

One of the most common ways to determine individual dominance ranks, and the linearity (i.e. transitivity) of dominance hierarchies under laboratory conditions, is by using the competitive exclusion (CE) task (i.e. tube test). The CE task was first designed by Lindzey *et al*.^14^, and later used in round-robin tournaments between cage-mates^15^, to assess which mice consistently retreat from others in a face-to-face conflict within a long and narrow tube. This task allows for clear scores of dominance; “win”, “lose”, or “tie”, which are difficult to quantify by home cage observations^15–17^. The task has been validated against other measures of dominance such as; home-cage behaviour, urine marking assay, food competition, and courtship vocalization^18–21^; although not all measures of dominance necessarily converge^15,22^. Nonetheless, the CE task may allow for characterization of dominance relationships and hierarchies within groups of mice housed under laboratory conditions.

Research assessing dominance hierarchies suggests that mice with different dominance ranks may vary in their behaviour and physiology. For example, more dominant mice often exhibit higher levels of exploratory behaviour when exposed to novel environments compared to less dominant mice^3,23–28^, while effects on behavioural measures of anxiety are inconsistent (SI Table 1)^3,21,25,29,30^. Studies relating basal glucocorticoid levels to dominance rank in laboratory mice also report inconsistent findings, but generally have found that dominant mice have higher levels of basal glucocorticoids compared to subordinate mice when housed in small groups (2–5 mice) for several weeks^4^. Body mass is frequently unrelated to dominance rank in laboratory mice^21^; although large differences (>30%) in body mass have been related to rank — with heavier mice being more dominant^31,32^. Thus, phenotypic variability in behaviour and physiology may exist between cages of laboratory mice depending on their rank within a dominance hierarchy.

Despite this theoretical and empirical evidence, it is generally unknown how dominance rank may relate to phenotypic variation within and between cages. We hypothesized that the social dominance rank of mice, housed under standard laboratory conditions, would be associated with phenotypic variation and would account for more variation than cage assignment. To test this hypothesis, we focused on three main predictions. First, mice should maintain linear (transitive) and stable dominance ranks across multiple weeks. Second, multiple measures of phenotype should consistently vary between mice of different dominance ranks. Third, social dominance rank should account for more variation in phenotype than cage assignment on multiple measures of phenotype.

To test these predictions, we assessed social dominance relationships within cages and related dominance ranks to measures of behaviour and physiology of male and female mice housed in same-sex groups under standard laboratory conditions. Dominance relationships were assessed three times, each spaced one week apart, using the CE task. Behaviour in a novel object test and elevated zero-maze, along with measures of stress physiology (faecal glucocorticoid metabolites) were taken to evaluate the relationship between dominance ranks and phenotype between cages.

## Materials and Methods

### Experimental Design

A total of 48 male and 45 female C57BL/6ByJ mice were used as subjects for this experiment, and were a subset of animals from a previous experiment comparing space allowance on measures of animal welfare (see SI Text 1 for further information, and the previous experiment)^33^. Mice for the current experiment were maintained in the same conditions as the previous experiment^33^; groups of 5, in either Makrolon Type 2 (225 × 167 × 140 mm) or Type 3 (382 × 220 × 150 mm) polypropylene cages for the entire experiment. Sex was counter-balanced across 3 experimental batches (see SI Table 2 for further information). The mice ranged in age from 6–16 months and were housed together for at least 23 weeks prior to testing. As aggressive behaviour peaks at 10 weeks of age, the use of relatively older mice increased the probability of observing stable dominance hierarchies (see hypothesis) compared to younger mice housed together for only a few weeks^34^.

**Figure 1:**
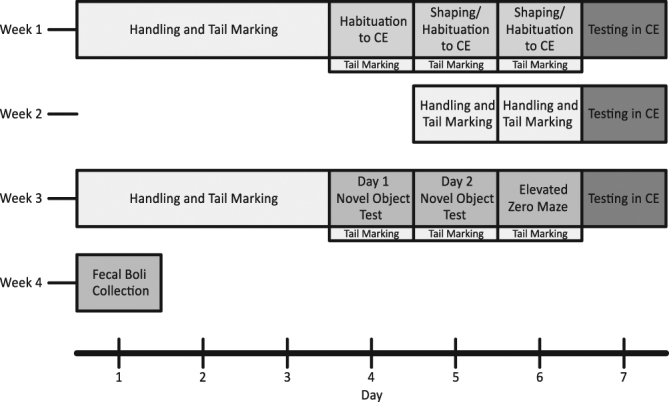
Experimental timeline for a single batch of mice.

The following outcome measures were collected: a) dominance rank based on competitive ability in the CE task; b) behaviour in a novel object task; c) behaviour on an elevated zero-maze; d) body mass; and e) faecal samples to determine concentration of basal glucocorticoid metabolites (Fig. 1).

### General Husbandry Procedures

All subjects were kept on a 12:12 light/dark cycle with lights on at 21:00. Temperature was maintained at 23 ± 1 °C and humidity ranged between 36 ± 22%. All cages contained wood shavings 2 cm deep (Lignocel® select), and animals had *ad libitum* access to standard rodent chow (Kliba Nafag #3430, Switzerland) and tap water. Cages, bedding, and water bottles were changed once every seven days in the dark phase under red light, and the body mass for each mouse was measured and recorded at this time. Cages were provided 10 to 11 grams of nesting material (Sizzle-Pet®), and half of the unsoiled nesting material was transferred at cage change from the used cage to the new cage as this has been shown to reduce the incidence of aggression^35^. All animals were ear tattooed one day after arrival for identification purposes.

### Ethical Statement

This study was carried out in accordance with the guidelines of the Swiss Animal Welfare Ordinance (TschV 455.1). It was approved by the Cantonal Veterinary Office in Bern, Switzerland (permit number: BE4/14).

### Competitive exclusion (CE) task

We used an adapted version of the CE task in a round-robin tournament between cage-mates^14,15,18,21^, with the addition of a vertical sliding door positioned in the middle of the tube to initiate the start of the trial^36^. Cages of mice were distributed between two experimenters (JV and JB), counterbalanced for sex and type of Makrolon cage. The order of testing of dyads was also randomized, using random.org, and no member of the previous pair was tested consecutively (see SI Text 2 and SI Fig. 9 for further information). Mice were tested across three batches, and the age of animals tested was counterbalanced between batches.

### Social Dominance Scoring

Social dominance ranks were determined following each CE tournament by calculating ordinal rankings and Normalised David Scores (DS; continuous). The dominance hierarchy within each cage during each week was determined by calculating Landau’s h, directional consistency, and steepness. Landau’s h measures the degree of linearity within the social hierarchy using ordinal rankings, and ranges from 0 (no linearity) to 1 (completely linear)^37^. Hierarchies were considered linear when Landau’s h was greater than 0.70, meaning all possible triads were transitive^38^. Directional consistency was calculated to assess the degree of wins compared to losses for each possible dyad in a group. Directional consistency scores ranged from 0 (symmetric relationship with equal wins and losses) to 1 (asymmetrical relationship with one individual winning all interactions)^39^; and these scores were used to compare relationships between ranks across week. Steepness was calculated by running a regression of the Normalised DS against the rank order of the group members to measure the absolute differences in dominance rank between adjacently ranked mice. The steepness score ranged from 0 (minimal differences in dominance rank) to 1 (maximal differences in dominance rank)^40^ (see SI Text 3 for further information).

### Novel Object Test

The novel object test was conducted in a square arena with climbable novel objects, and involved a single habituation trial, three familiarization trials, and a single test trial^41–43^ (see SI Text 4 and SI Fig. 10 for further information). This test measures object exploration with the expectation of decreased exploration of objects during familiarization trials (habituation), and renewed exploration following the introduction to a novel object (dishabituation). The outcome variables of interest were total distance travelled, duration of proximity to the objects (proportion of time the center-point of the mouse was within 7.75 cm of an object), and a novel object discrimination index (DI); equation (1). The DI was only calculated for the test trial.

1 $$DI=\frac{{\rm{duration}}\,{\rm{of}}\,{\rm{proximity}}\,{\rm{to}}\,{\rm{novel}}\,{\rm{object}}}{({\rm{duration}}\,{\rm{of}}\,{\rm{proximity}}\,{\rm{to}}\,{\rm{novel}}\,\text{object})+(\text{duration}\,{\rm{of}}\,{\rm{proximity}}\,{\rm{to}}\,{\rm{familiar}}\,\text{object})}$$

### Elevated Zero-maze

The elevated zero-maze used in this study was similar to the original test described by Shepherd *et al*.^44^. This test measures exploration of open (unprotected) sectors of a circular runway compared to closed sectors (protected by high walls). Mice that spend more time in the open arms than closed arms explore the apparatus more; a sign of low levels of anxiety in this test situation^45^. The outcome variables of interest were total distance travelled, time spent in the open sectors, and the frequency of entering the open sectors (see SI Text 5 for further information).

### Glucocorticoid metabolites

Faecal boli samples were collected the day immediately following the last CE test in the dark phase, under red light from 9:00–13:00. Mice were individually housed in isolation with fresh bedding, food, and water. After isolation, mice were placed back in their home-cage with their cage-mates. Individual boli samples were then collected from each cage, stored at −20 °C, and later processed (JV and RP) according to the well-established method described by Touma and colleagues^46^. The outcome variable of interest was the concentration (ng/0.05 g faeces) of glucocorticoid metabolites (sharing a 5α-3β, 11β structure) (See SI Text 6 for further information).

### Statistical analyses

All statistical analyses were performed with R (version 3.4.3). Assumptions of normality of error distribution, and homogeneity of variance were examined graphically. Based on these inspections, no transformations of data were performed. Because it is well recognized that males generally show higher levels of agonistic behaviour than females^34,47^, males and females were analysed separately. Stability of individual rank was measured using Spearman correlations following the method of Oliveria & Almada^48^, where r_s_ >0.70 indicates stability. Stability was also determined by the frequency of changes in individual rank across week. Spearman correlations were used to compare variables with ordinal rank, and Pearson correlations were used to compare variables with Normalised DS (continuous rank). Analyses of change in the stability of hierarchy were performed using a univariate general linear model with week as a fixed effect. Linear mixed-effects models (‘lmer’ function with ‘bobyqa’ optimization) were used to test whether there was more variation between dominance ranks compared to between cages. Here, individual mice (48 males and 45 females) were nested within cages (10 male cages and 9 female cages). Cage assignment (number used to identify which cage each mouse was associated with) was a random effect, while Makrolon Cage Type (2 levels; Type 2 and Type 3) and ordinal rank (5 levels; alpha, beta, etc.) were fixed effects. Age of mouse was also included as a covariate in the model. A likelihood ratio test (‘rand’ function) was performed to estimate whether the fitted model was improved with the inclusion of the random effect “cage assignment”. P-values < 0.05 were considered statistically significant.

### Data and materials availability

All data needed to evaluate the conclusions in the paper are present in the paper and/or Supplementary Materials. Additional data related to this paper may be requested from the authors

## Results

### Social Dominance Hierarchies and Ranks

**Figure 2:**
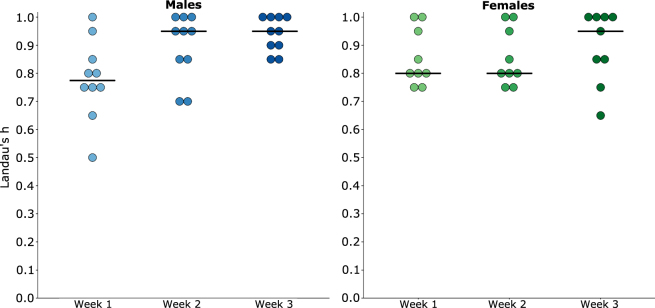
Change in linearity (Landau’s h) from weeks 1–3 for cages of males and females. Bars represent medians and scores higher than 0.70 represent transitive hierarchies.

Social dominance hierarchies and ranks were determined for each cage following each round-robin tournament in the CE task. The linearity of each hierarchy was measured using Landau’s h index, which provides the degree to which a group of mice within a cage adheres to a completely linear hierarchy — with all transitive triads. We found that social dominance hierarchies significantly increased in linearity, from weeks 1 to 3 for cages of males (F_2,9_ = 5.451, p = 0.01), but found no evidence of changes in linearity for cages of females (F_2,8_ = 0.367, p = 0.697). We hypothesized that all cages would have a linear hierarchy with no intransitive triads at each measured week; however, this effect was only present in week 3 for cages of male mice (10 out of 10 groups) (Fig. 2). Female mice had the highest frequency of linear hierarchies each week, but had the highest levels of linearity on week 3 compared to any other weeks; with only 1 non-transitive group. Therefore, as measured by Landau’s h, dominance hierarchies were the most linear at week 3; indicating that each mouse had a unique rank within a cage.

To further understand the organization of the dominance hierarchy, a secondary measure was then calculated, namely the steepness index of the hierarchy. The steepness index reflects the degree to which mice similar in rank, within a cage, are also similar in their proportion of wins. There was no evidence that steepness changed from weeks 1 to 3 for males or females (SI Fig. 1), and there was a wide range in steepness scores in the 3^rd^ week (Males: range 0.48–0.78; Females: range 0.48–0.76) when groups were most linear (SI Fig. 2). This wide range suggested that the mice of some cages had similar proportions of wins despite having linear hierarchies with unique dominance ranks.

Next, we evaluated the stability of dominance rank, as increases in linearity across the 3 weeks and similar proportions of winning between mice within a group suggested that rank within a cage was not unique. Ordinal dominance ranks for each group were calculated by ranking the total wins per group member in descending order for each tournament day (see SI Text 3 for more details). The stability of ordinal dominance ranks was calculated using a Spearman correlation for individual mice across the 3 weeks of CE testing (Males: r_s_ = 0.712; Females: r_s_ = 0.782) — correlations greater than 0.70 were considered to reflect stable ranks^48^. Although these correlations provided evidence that ranks were stable across the 3 weeks of testing, 66.6% of male mice and 71.1% of female mice changed their individual ordinal rank at least once from weeks 1 to 3. For male mice, stability increased when comparing weeks 2 and 3; with r_s_ = 0.847 and 37.5% of males changing rank from week 2 to 3. For female mice, the difference in stability was less pronounced; with r_s_ = 0.748 and 46.6% of females changing rank from week 2 to 3. Ordinal ranks were highly correlated with Normalised DS, which reflects the total proportions of wins and losses (see SI Table 3).

**Figure 3:**
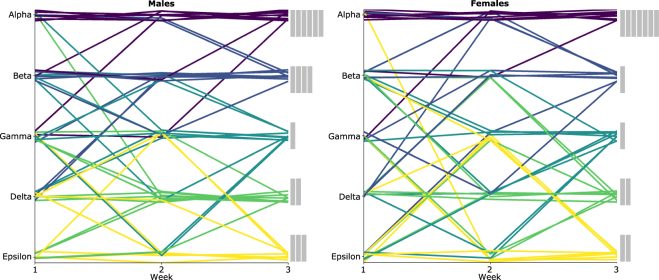
Ordinal dominance rank from weeks 1–3 for groups of males and females. Individuals are coloured depending on their assigned rank at week 3. Histograms on the right-side of the line-graph show the number of mice who kept a stable rank for the entire 3 weeks; each bar represents one mouse.

Despite frequent changes in ordinal rank, alpha ranks were the most stable compared to other ranks for both males (Weeks 1 to 3: 6 out of 10; Weeks 2 to 3: 8 out of 10) and females (Weeks 1 to 3: 7 out of 9; Weeks 2 to 3: 8 out of 9) (Fig. 3). Furthermore, mean directional consistency scores, comparing the consistency of wins for each trial per ordinal rank pair, showed that alpha males and females had the most unidirectional relationships by week 3 (Males: range 0.812–0.938; Females: range 0.944–1) compared to other social dominance ranks (Males: range 0.562–0.812; Females: 0.333–0.812); see SI Fig. 3.

Overall, dominance hierarchies were more stable and linear between weeks 2 to 3 compared to weeks 1 to 2. Furthermore, both males and females had the highest frequency of linear hierarchies in week 3. Therefore, ordinal rank during week 3 was used to assess the relationship between measures of dominance relationships and variation in phenotype. Because ordinal rank during week 3 was significantly correlated with all other measures of dominance rank, and unique dominance ranks were required for assessing our hypotheses relating to variation between dominance ranks, other measures of dominance relationships were not used in further analyses.

### Novel Object Test

Exploration in the novel object test was measured across three 6-minute familiarization trials followed by a 6-minute novel object exposure trial to test whether ordinal rank or cage assignment was related to novel object exploration. Across the three familiarization trials, females but not males differed in their duration of time in proximity to the objects (Male: F_2,144_ = 1.482, p = 0.231; Female: F_2,135_ = 3.202, p = 0.044); however, *post hoc* tests suggested this difference was due to an increase in object exploration from trial 1 to trial 2 (SI Fig. 4). As expected, total distance travelled across the three familiarization trials decreased for males and females (Male: F_2,144_ = 18.174, p < 0.001; Female: F_2,132_ = 16.385, p < 0.001). For the novel object test, there was no evidence that males and females spent more time near the novel object compared to the familiar object (Mean DI: Male: 0.55; Female: 0.49).

**Figure 4:**
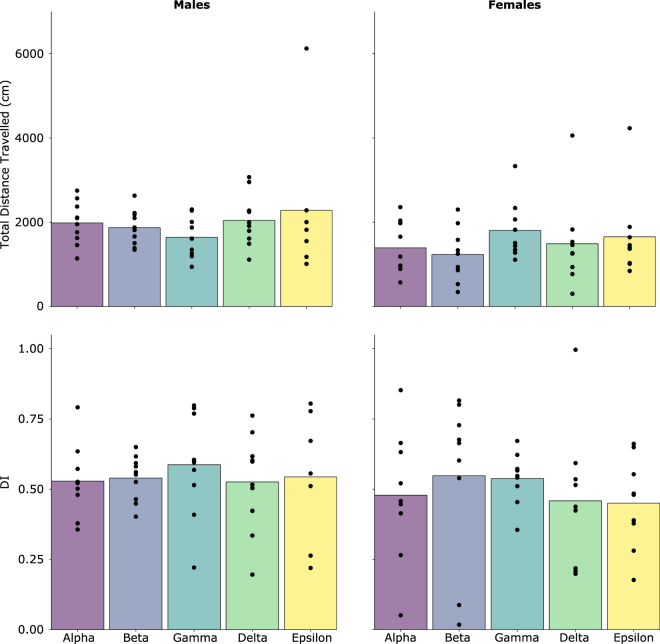
Relationship between Ordinal Rank at week 3 and exploratory behaviour in the novel object test for males and females.

There was also no statistically significant effect on variation in total distance travelled during the novel object test for males or females in relation to ordinal rank measured during week 3 (Male: F_4,34_ = 0.715, p = 0.588; Female: F_4,32_ = 0.780, p = 0.547) (Fig. 4) or cage assignment (Male: *X*^2^ (1, N = 48) = 0.010, p = 0.900; Female: *X*^2^ (1, N = 45) = 0.239, p = 0.600) (SI Fig. 5). Furthermore, there was no evidence that variation in the DI of the novel object for males or females was accounted for by ordinal rank on week 3 (Male: F_4,34_ = 0.235, p = 0.917; Female: F_4,38_ = 0.381, p = 0.821) or cage assignment (Male: *X*^2^ (1, N = 48) = 0.160, p = 0.700; Female**:** *X*^2^ (1, N = 45) = −1.6 × 10^−14^, p = 1.000).

### Elevated Zero-Maze

Exploration of the elevated zero-maze was measured for 5 minutes on the day immediately following the novel object test to measure whether cage assignment or ordinal rank was related to time spent in the open arms, frequency entering open arms, and total distance travelled in the elevated zero-maze. Overall, mice spent less time in the open arms compared to the closed arms (t = −23.898, p < 0.001). There was large individual variation in both males and females with respect to time spent in the open arms, frequency of entering open arms, and total distance travelled; see SI Table 4.

**Figure 5:**
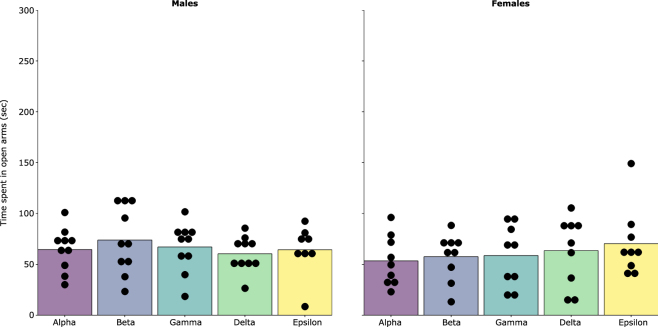
Relationship between exploratory behaviour in the elevated zero-maze and ordinal dominance rank on week 3 for males and females.

**Figure 6:**
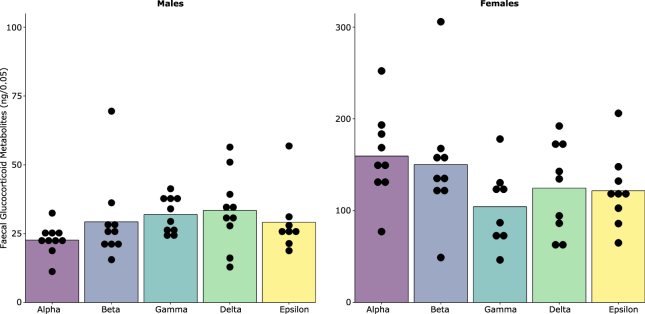
Relationship between basal faecal glucocorticoid metabolite levels and ordinal dominance rank on week 3 for males and females. Note; there are different axes for males and females because males and females are not directly comparable using this method of quantification, See SI Text 6.

There was no evidence that ordinal rank was associated with time spent in the open arms (Ordinal Rank: Male: F_4,34_ = 0.626, p = 0.647, Female: F_4,38_ = 0.524, p = 0.719) There was evidence, however, that cage assignment was associated with time spent in the open arms, for males only (Cage assignment: Male: *X*^2^ (1, N = 48) = 9.580, p = 0.002, Female: *X*^2^ (1, N = 45) = 0.723, p = 0.400) (Figs 5 and 6). There was no evidence that ordinal rank and cage assignment was associated with frequency of entering open arms (Ordinal Rank: Male: F_4,34_ = 0.142, p = 0.965, Female: F_4,38_ = 0.449, p = 0.772; Cage assignment: Male: *X*^2^ (1, N = 48) = 2.830, p = 0.090, Female: *X*^2^ (1, N = 45) = 0.450, p = 0.500), or total distance travelled (Ordinal Rank: Male: F_4,34_ = 0.927, p = 0.456, Female: F_4,38_ = 0.619, p = 0.652; Cage assignment: Male: *X*^2^ (1, N = 48) = 1.460, p = 0.200, Female: *X*^2^ (1, N = 45) = 0.299, p = 0.600). See SI Text 7 for *post hoc* exploratory analyses on stability of rank and exploratory behaviour in the elevated zero-maze.

### Glucocorticoid metabolites

Faecal samples for each mouse were collected following the third CE test. Similar to exploratory behaviour in the elevated zero-maze, large individual variation in both males and females was observed, irrespective of dominance rank and cage assignment. There was no evidence that levels of faecal glucocorticoid metabolites (sharing a 5α-3β, 11β structure) were associated with ordinal rank measured on week 3 (Male: F_4,34_ = 1.583, p = 0.199; Female: F_4,32_ = 1.918, p = 0.1299) (Fig. 6) or cage assignment (Male: *X*^2^ (1, N = 48) = 5.68 × 10^−14^, p = 1.000, Female: *X*^2^ (1, N = 45) = 5.68 × 10^−14^, p = 1.000) (SI Fig. 7).

### Body Mass

**Figure 7:**
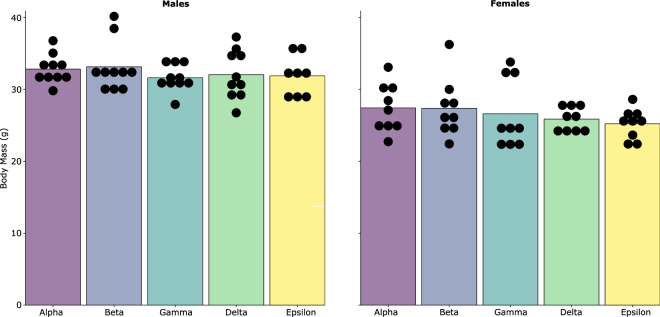
Relationship between body mass and ordinal dominance rank on week 3 for males and females.

Linear mixed models indicated there was no evidence that body mass was associated with ordinal rank measured on week 3 (Male: F_4,34_ = 0.607, p = 0.660; Female: F_4,32_ = 1.272, p = 0.299) (Fig. 7) or cage assignment (Male: *X*^2^ (1, N = 48) = 0.058, p = 0.800, Female: *X*^2^ (1, N = 45) = 5.68 × 10^−14^, p = 1.000) (SI Fig. 8).

## Discussion

The overall aim of this study was to determine if social dominance ranks are associated with phenotypic variation and account for more variation than cage assignment^1,6^. We predicted that mice would maintain linear and stable dominance ranks, that phenotype would consistently vary between mice of different dominance ranks, and that dominance rank would account for more variation than cage assignment. In contrast to these predictions, dominance ranks changed with time, but hierarchies were most linear and most stable on the 3^rd^ week of testing. There was little evidence to suggest that mice of different dominance ranks, or that mice from different cages, differed in our measures of behaviour and physiology.

### Social dominance hierarchies and ranks

We determined the social dominance ranks of mice within cages by measuring dominance hierarchies using the CE task in a round-robin tournament once per week for 3 weeks. All groups of male mice, and 7 out of 9 groups of female mice had linear social dominance hierarchies. This result was expected for groups of male mice^20,49^, and adds to the limited research on dominance hierarchies in female mice — housed under laboratory conditions^36,50^.

Linearity of dominance hierarchies increased in groups of male mice from week 1 to 3, while linearity did not differ across weeks in groups of females. Given that all groups were expected to have stable dominance hierarchies because they maintained their group housing cohorts for at least 23 weeks, the most likely explanation for an increase in linearity in male mice is that they were unfamiliar with the CE task on week 1 and were winning and losing a similar proportion of trials. Thus, ordinal rank measured at week 1 may have been less representative of the true rank of each mouse. This conclusion is supported by a recent review which suggested that the CE task should be conducted on at least 3 consecutive days, or until mice show a stable rank position^21^. Groups of mice are expected to increase in linearity of the dominance hierarchy over a period of 3 weeks as the dominance hierarchy emerges^51^. The mice in this experiment, however, had been housed together for at least 23 weeks prior to the first week of testing, and were tested at different ages. Therefore, this increase in linearity is unlikely to be the result of an emerging dominance hierarchy.

Both male and female mice frequently switched ranks from weeks 1 to 3, however, the frequency of switching decreased in weeks 2 to 3. This indicated that the mice had unstable or unclear rank positions; however, it also provides secondary evidence that mice were unfamiliar with the CE task during the first week of testing. This increase in rank stability from weeks 2 to 3, suggested that performance in the CE task became more consistent across time. Increased consistency may indicate that learning might be taking place — a key element to dominance behaviour, and maintenance of a dominance hierarchy^52^. The CE task is intended to reflect the learned dominance relationships associated with the home-cage, however, the observed increases in the consistency of wins and losses in our CE task hints that the observed dominance relationships may be specific to the CE task. For example, repetitive testing in the CE task can often result in “trained” winners and losers independent of dominance behaviour in the home-cage^52^. Furthermore, previous researchers studying dominance have noted that dominance relationships may depend on spatial context, and are different when groups interact in different contexts like the CE task^17,22^. We cannot examine this hypothesis, on spatial context, further because we did not assess dominance relationships in the home-cage. However, based on the observed data we cannot discount that the pattern of outcomes in the CE task may reflect learned dominance behaviour disparate from home-cage dominance behaviour.

Although mice had linear hierarchies, indicating unique dominance ranks, other measures of dominance suggested mice had more despotic than linear dominance hierarchies. Despotic hierarchies are composed of an alpha that wins a majority of interactions and subordinates, which have no clear ranking between one another^10^. Measures of steepness and stability of ordinal ranks suggested that mice ranked alpha had unique ranks with the highest proportion of wins compared to all other mice within a group. Furthermore, measures of normalised DS suggested that all mice except alpha had similar proportions of winning. Therefore, it is probable that groups in our study were primarily organized into a despotic hierarchy, and ranks observed below the alpha were an artefact of the limited number of trials and winner-loser effects in the CE task. However, without data on home-cage behaviour, this conclusion remains speculative at best.

Rank positions might have also been unstable or unclear because groups were mostly composed of 5 mice per cage in standard laboratory housing. As group size increases, the number of possible dyadic relationships within a group also increases, which increases the probability that a mouse will change rank or show a different rank across time. For example, groups of 3 mice only have 3 possible dyadic relationships, while groups of 5 mice have 10 possible dyadic relationships. Furthermore, several studies have suggested that increasing group size often leads to more aggression, which is a sign of unstable or unclear dominance ranks^51,53,54^. However, recent findings also suggest that groups of 12 male mice can maintain a relatively stable dominance hierarchy^49,55^. These mice, however, were grouped in early adulthood in complex housing with tubes, nest-boxes, and monopolizable resources — in stark contrast to standard laboratory conditions. Thus, given our current dataset and the current literature, instability and similarity of ranks other than alpha mice may be due to a combination of group size, cage size, and cage complexity.

Although dominance ranks were not always unique or stable between mice, except for mice ranked as alpha, dominance ranks were most stable between weeks 2 to 3. Groups of mice were also most linear at week 3, despite other measures suggesting a more despotic organization. The observed level of stability and linearity between weeks 2 to 3 is in agreement with recent research using the CE test^18–21,36^. Therefore, ordinal rank on week 3 was used to assess the relationship between dominance rank and phenotype because ordinal rank was the most stable at this time point.

### Phenotypic variation within and between social dominance ranks

Phenotypes of all mice were measured to compare phenotypic variability between dominance ranks and between cage assignment. High levels of individual differences were observed for all outcome measures, but neither dominance rank nor cage assignment systematically accounted for this variation.

It is unclear why dominance rank was unrelated to exploratory behaviour or glucocorticoid metabolites between cages. Previous studies, on exploratory behaviour and glucocorticoids, have found differences between dominant and subordinate mice^3,23,24,27–29,54,56–58^, while other studies have reported no differences^3,20,25,57–59^ (See SI Table 1, and *c.f*.^4^). One possible reason that dominance rank was unrelated to any of the measured outcomes may simply be that many mice had an unstable, or unclear, dominance rank. We predicted that all mice would have stable hierarchies with clear dominance ranks. Because mice frequently switched rank throughout the three weeks, it is possible that their assigned rank in week 3 was not an accurate characterization of their social status. A more appropriate characterization would consider their general position within the hierarchy and frequency of rank switches. Unfortunately, this complex method of assigning social status was limited by our frequency of measuring dominance^49^, and was potentially confounded by our observation that rank in the first week of CE testing was in part due to unfamiliarity with the task. Furthermore, the possibility of learned dominance rank in the CE task might limit its construct validity as a general test of dominance rank, because ranks derived from this task might not be a good representation of dominance ranks in the home-cage.

Within sex, mice had a similar body mass, unrelated to dominance rank or cage assignment. Some studies have reported that a relationship between dominance rank and body mass exists when differences in body mass are greater than 30% within cages^31^. Because dominance rank may be related to body mass, similarities in body mass within cages may have further contributed to unstable or unclear dominance relationships. Thus, future studies could investigate whether increasing differences in body mass within cages increases stability or unidirectionality of dominance relationships within groups of mice compared to groups similar in body mass.

Overall, our analyses indicate that there is considerable individual variation in behavioural and physiological measures in mice. This variation in phenotype, however, was not systematically accounted for by dominance rank, or by cage assignment — albeit cages of males were statistically different for a single phenotypic measure; time spent in the open arms of the elevated zero-maze. Studies investigating the individuality of mice suggest that multiple factors such as; minisatellite variation, intrauterine position, early nutrition, differences in maternal care, or litter effects may contribute to individual differences^6,60^. Such factors could potentially have masked the effect of dominance ranks on the observed phenotypic variation.

## Conclusion

The overall aim of this study was to assess how social dominance rank and cage assignment relate to variation in phenotype. A large proportion of mice switched rank over the course of 3 weeks, making the assignment of social dominance ranks and the ability to assess rank related variation difficult. Furthermore, repeated measurement of individual ranks suggested that individual ranks within a cage may not have been unique, and that groups formed more despotic than linear hierarchies. Using linear mixed models, with social dominance rank as a fixed effect and cage assignment as a random effect, we found little to no evidence to suggest that dominance rank or cage assignment are associated with phenotypic variation in exploratory behaviour in the elevated zero-maze and in a novel object test, concentrations of faecal glucocorticoid metabolites, and body mass in males or females, respectively. A single measure of time spent in the open arms of the elevated zero-maze was associated with cage assignment, but only for males. It is unclear whether this single difference is artefactual, particularly given that none of our other phenotypic measures varied with cage assignment. More research is necessary to determine how stable and unstable social dominance relationships are related to phenotypic variability in common behavioural tests, and whether part of the large phenotypic variability that exists within cages of mice can be explained by dominance rank. Future studies should compensate for heterogeneity in dominance stability by increasing sample size, include other strains of mice, and different group sizes.

## Electronic supplementary material

Supplementary Information
