# Supplementary material for: Phenotypic variability between Social Dominance Ranks in laboratory mice
Source: Sci Rep. 2018 Apr 26;8:6593. doi: 10.1038/s41598-018-24624-4 (PMC5920077; doi:10.1038/s41598-018-24624-4)

Phenotypic variability between Social Dominance Ranks in laboratory mice

Justin A. Varholick[1*], Jeremy D. Bailoo[1], Rupert Palme[2], and Hanno Würbel[1]

[1]Division of Animal Welfare, Veterinary Public Health Institute, University of Bern, Bern, Switzerland
[2]Department of Biomedical Sciences, University of Veterinary Medicine, Vienna, Austria

Supplementary Information:
Number of pages: 15
Number of figures: 10
Number of tables: 4

SI Text 1: Methods, Subjects

Subjects and Design (cont.)

All mice in this experiment were derived from a previous housing experiment [33] that compared the phenotypes of mice housed in 3 different cage-sizes and in 3 different group-sizes (3, 5, 8 mice) using both males and females of two strains: C57BL/6J and BALB/c – no other treatments were administered. For the current experiment, we only used C57BL/6byJ mice housed in groups of 5 in cages of two different sizes (Makrolon Type 2 and 3). The results of that study indicated that there were no statistically significant differences in phenotypes between Type 2 and 3 cages with mice housed in groups of 5. In the previous study a range of measures, including a) Growth (food and water intake, body mass); b) Stress Physiology (glucocorticoid metabolites in faecal boli at the cage level); and c) Home-cage behaviour (activity, stereotypic behaviour) were assessed for all mice, while only one randomly selected mouse from each cage was tested for d) Emotionality (open field behaviour); and e) Perseveration (two-choice guessing task). Given that these tests were quick and non-invasive it is unlikely that there were any carry-over effects between these two experiments, or any differences between the focal and non-focal mice of the previous study that go beyond other individual differences in previous experience (e.g. those induced by differences in social dominance status).

Two males from separate cages were euthanized before testing due to severe wounding, leaving 2 groups with 4 males per cage and 8 groups with 5 males per cage. A single cage of females was also euthanized before testing due to severe alopecia, leaving a total of 9 groups of 5 females per cage. Cages were pseudo-randomly distributed across 3 experimental batches according to Makrolon Type and age (see SI Table 1). Although two different Makrolon Type cage sizes were used in this study, we posited that the stability and linearity of dominance rank would be independent of Makrolon Type cage. We also posited that although the age of the mice ranged from 6-16 months-old, all groups of mice should have a stable dominance hierarchy, if indeed one was present.

SI Text 2: CE task

Apparatus

The apparatus consisted of two grey polycarbonate boxes (10 x 7 x 8 cm) connected together by a clear 25cm long Plexiglas tube. The tube diameter was just wide enough for one mouse to fit through, narrow enough for two mice to not pass one another, and difficult for a single mouse to turn 180 degrees. Most mice (77%) were tested in a 2.5 cm diameter tube; however, if a mouse was too large and had difficulty traversing the tube, testing occurred in a 3cm diameter tube (determined during shaping). A vertical sliding door was positioned in the middle of the tube to initiate the start of the trial (SI Fig. 9).

Procedure

On the first day each mouse was habituated to the apparatus by placing them in one of the boxes with a randomly selected cage-mate and allowing them to explore for 5 minutes. On the next two days mice were individually shaped to cross the tube when placed in the apparatus across four trials daily. A single trial involved placing the mouse in a box, and gently nudging them to cross the tube twice if they did not cross the tube voluntarily. Trials were counter-balanced for side of placement. Each cage participated in a predetermined round-robin tournament on each test day. For each trial, two mice were selected from a single cage and simultaneously placed at opposite ends of the apparatus (one in each hand). Once the two mice met in the middle, the door was opened vertically, and the trial was begun. The first mouse to withdraw from the tube by placing at least the two rear paws on the floor of the starting box was recorded as the “loser” of the trial [18], and the other mouse, the “winner”. If the two mice withdrew at the same time, a “tie” was recorded for each mouse – there were 10 ties in total out of 2,184 trials. This process was repeated four consecutive times for each pair of mice within a cage with removal of the pair between trials [18] – 40 separate trials for groups of 5, and 24 separate trials for groups of 4.

All habituation and testing were done in the dark phase under red light, and the apparatus was wiped down with 70% ethanol immediately after pairs of mice were returned to their home-cage. One

cage only participated in 36/40 trials in the 3rd week of testing because one mouse was averse to entering the tube after getting its snout stuck on the side of the door during initiation of the trial.

SI Text 3: Social dominance calculations

Calculating social dominance scores

Ordinal ranking was determined by ranking the total wins for each group member in descending order. If a pair of group members had the same number of total wins at the end of a tournament, the group-member with more wins when paired against the other group member with the same number of wins in the CE task, was given the higher rank. Ordinal ranking provided unique ranks for each group member. Normalised David's Score (DS) calculated the proportion of wins and losses (competitive behaviour) of each individual, correcting for the number of total interactions and number of animals in a group [37]. Normalised DS provided a continuous ranking and ranged from 0 to 4; with 0 representing least dominant and 4 representing most dominant.

Social dominance matrices

All dominance ranks, and the organization of the dominance hierarchy metrics were determined by making binary and non-binary win-loss matrices using wins, losses, and ties. For binary win-loss matrices; wins were assigned a score of 1, losses of score of 0, and ties a score of 0.5 [55]. For non-binary matrices; wins were assigned a score of 1, losses a score of 0, and ties a score of 0.5. Summed wins, losses, and ties for each dyad were then organized into a binary and a non-binary win-loss matrix for each tournament using the compete package v0.1 in R [55]. Ordinal ranking, Normalised DS, Landau's h, and directional consistency were also calculated using the matrices and the compete package v0.1 in R [55]. Steepness was calculated using the steepness package v0.2-2 in R [37].

I&SI scoring

Inconsistencies and strengths of inconsistencies (I&SI) ranking was also calculated using binary win/loss matrices. The I&SI ranking is based on an algorithm that re-organizes the binary matrix assuming the hierarchical organization is as linear as possible [55].

SI Text 4: Novel Object Test

Apparatus

Four separate apparatuses were used for this test. Each apparatus was a square arena (44 x 44 x 44 cm) made of grey polycarbonate with an opaque white polycarbonate floor. The four arenas were positioned in a square under a centred camera, centred low lumen light (15 lux), and two additional red lights in the midline. The video camera was connected to a computer for live recording with Ethovision XT version 11®. The objects used in the experiment were a bright green polycarbonate box (6.75cm x 6.75cm x 5.5cm) with large circular bumps on each side, and a dull white polycarbonate cylinder (6.75cm x 6.75cm x 5.5cm) with ridges on 4 opposing sides (SI Fig 10). All objects were weighted to prevent the animal from moving them. Ethovision recorded the center-point of the mouse throughout the trial.

Procedure

The test took a total of 2 days, and mice were run in a predetermined randomized order within cage. On the first day (habituation), each mouse was placed with its head facing the wall of the open field facing away from where the objects would sit for familiarization and test trials. The mouse was habituated to the empty open field for 6 minutes. On the second day (familiarization trials 1-3 and test), each mouse underwent three 6-minute familiarization trials, and a 6-minute test trial, with 3-minute intervals between each trial. During the intertrial intervals, familiar mice were placed together in a holding cage. For each familiarization trial mice were placed individually with their head facing the wall of the open field opposite of two predetermined randomized identical objects which were positioned 7.75 cm away from two perpendicular walls. For the test trial mice were placed with their head facing the wall opposite of a familiarized object and a predetermined randomized unfamiliar (novel) object. The side the novel object was presented on was counterbalanced across test subjects. To control for possible odour cues the objects and floors were cleaned with 70% ethanol, and multiples of each object were used such that no single mouse was exposed to a specific object more than once. All objects were wiped down after each trial.

SI Text 5: Elevated zero-maze

Apparatus

The apparatus consisted of one grey polycarbonate elevated zero-maze with an outer diameter of 46 cm, a 5.5 cm wide runway, and 16 cm high walls, fixed on four 40cm tall legs. A video camera connected to Ethovision was centred directly above the zero-maze, and two 130 lumen bulbs (40 lux) were positioned in the midline on opposite sides allowing for no shadows to be present across the entire apparatus. Ethovision recorded the center-point of the mouse throughout the trial.

Procedure

Testing consisted of one 5-minute trial per mouse, and mice were tested in a predetermined randomized order within each cage. The mouse was placed facing one of the closed sectors of the maze, counterbalanced for side between mice. After the 5 minute trial the mouse was returned to its home-cage and the apparatus was wiped down with a 70% ethanol solution.

SI Text 6: Glucocorticoid metabolites

Faecal boli were collected once in the dark phase under red light (c.f., Fig. 7). Starting at 9:00, mice from each cage were isolation housed in a Type 2 cages with with wood shavings (Lignocel® select) 0.5 cm deep, 3-5 g of food and *ad libitum* tap water. Because the gastrointestinal transit time for corticosterone metabolites in mice has been determined to be between 4-12 hours [46] we restricted collection of faeces to a maximum of 3-3.5 hours after isolation housing, to reduce the potential confounding influence of the stress of isolation housing on measured metabolite concentration. A minimum of 10 boli per mouse was collected. Samples were immediately frozen at -20°C and later blindly processed (JV and RP) according to the method described by Touma and colleagues [46]. A total of 93 samples were processed.

Female mice typically have higher faecal glucocorticoid metabolites compared to males, which is related to sex differences in corticosterone metabolism. The enzyme immunoassay for quantification of metabolites exhibits higher cross reactivity with metabolites secreted by females than males (Touma, C., Sachser, N., Möstl, E. & Palme, R. Effects of sex and time of day on metabolism

and excretion of corticosterone in urine and feces of mice. Gen. Comp. Endocrinol. 130, 267–278 (2003)).

SI Text 7: Stability of rank and phenotype exploratory analyses

Although rank at week 3 was determined to be the most suitable rank for measuring the relationship between dominance rank and phenotype, 37.5% of males and 46.6% of females changed ranks from weeks 2 to 3. We originally hypothesized that all cages would have stable ranks; however, this was not the case. Because two groups; stable and unstable, emerged from what we thought was one group, and because stability was somewhat necessary for our hypothesis on dominance rank and phenotype, we conducted exploratory analyses comparing stable and unstable groups. Mice were considered stable if they maintained their rank from weeks 1 to 3. Regardless of stability, we found that males explored the elevated zero-maze significantly more than females ($F_{2,9}$=5.674, p = 0.019). When only comparing stable alphas we found that this effect remained ($F_{1,13}$=7.501, p = 0.019). However, when comparing all mice except the stable alphas we found that there was no difference between males and females ($F_{1,80}$=2.655, p = 0.107). This suggests that the stable alphas might have been responsible for the observed sex difference.

SI Table 1

Relationship between behaviour and dominance rank

Behaviour	Test	Relationship between Rank and Behaviour	Sex	Group size	Strain	Citation
Exploratory	Home-cage activity	Dominant > Subordinate	M	2	Swiss-Webster	(24)
	Hole Board	Dominant = Subordinate	M	10	NIH Swiss	(25)
		Dominant > Subordinate	M	5	NIH Swiss	(3)
Anxiety-related	Open Field	Dominant > Subordinate	M	3	CD-1	(23)
		Dominant = Subordinate	M	4	C57BL/6J	(20)
		Dominant = Subordinate	M	4	C57BL/6J	(28)
		Dominant = Subordinate	M	4-5	C57BL/6J	(26)
		Dominant = Subordinate	M	4	C57BL/6J	(27)
	Elevated Plus Maze	Dominant > Subordinate	M	3	CD-1	(23)
		Dominant < Subordinate	M	10	Swiss-Webster	(29)
		Dominant = Subordinate	M	10	NIH Swiss	(25)
		Dominant = Subordinate	M	5	NIH Swiss	(3)
		Dominant > Subordinate	M	4	C57BL/6J	(27)
		Dominant < Subordinate	M	4	C57BL/6J	(28)
	Porsolt's Swim Test	Dominant > Subordinate	M	10	NIH Swiss	(25)
		Dominant > Subordinate	M	5	NIH Swiss	(3)
		Dominant < Subordinate	M	4	C57BL/6J	(28)
Cognitive	Lashley III Maze	Dominant > Subordinate	M	3	CD-1	(23)
	Morris Water Maze	Dominant = Subordinate	M	3	CD-1	(23)
	Odor Discrimination	Dominant = Subordinate	M	3	CD-1	(23)
Sensory/Motor	Balance Beam	Dominant = Subordinate	M	3	CD-1	(23)

SI Table 2

Number of mice per batch, across Age and Sex

			Age (in weeks)				
	Sex	n	55	50	36	31	26
Batch 1	Male	14	5	4	/	/	5
	Female	15	5	/	/	5	5
Batch 2	Male	15	/	/	5	5	5
	Female	15	/	5	5	/	5
Batch 3	Male	19	4	5	5	5	/
	Female	15	5	/	5	5	/
Totals	Male	48	9	9	10	10	10
	Female	45	10	5	10	10	10

Cells shaded grey indicate Type 3 cage-size

SI Table 3

Pearson correlations for ordinal and I&SI rankings, and Spearman correlations for categorical and DS for males and females across weeks.

**Sex**			**Week**	**r**	**p**
Male	Ordinal	I&SI	1	0.935	< 0.001
			2	0.95	< 0.001
			3	0.995	< 0.001
	Ordinal	DS	1	-0.876	< 0.001
			2	-0.897	< 0.001
			3	-0.886	< 0.001
Female	Ordinal	I&SI	1	0.961	< 0.001
			2	0.938	< 0.001
			3	0.978	< 0.001
	Ordinal	DS	1	-0.953	< 0.001
			2	-0.932	< 0.001
			3	-0.965	< 0.001

SI Table 4

Range of exploration in elevated zero-maze.

Sex	Variable	Min	Max	Mean	SD
Male	Time Spent in Open Arms	8.56	113.96	66.13	23.97
	Frequency of Entering Open Arms	3	33	18.4	6.78
	Total Distance Travelled	593.48	1653.61	1128.54	242.89
Female	Time Spent in Open Arms	13.2	149.12	60.66	28.69
	Frequency of Entering Open Arms	5	32	17.56	7.36
	Total Distance Travelled	598.99	1826.93	1003.48	263.4

SI Fig. 1: Steepness across weeks

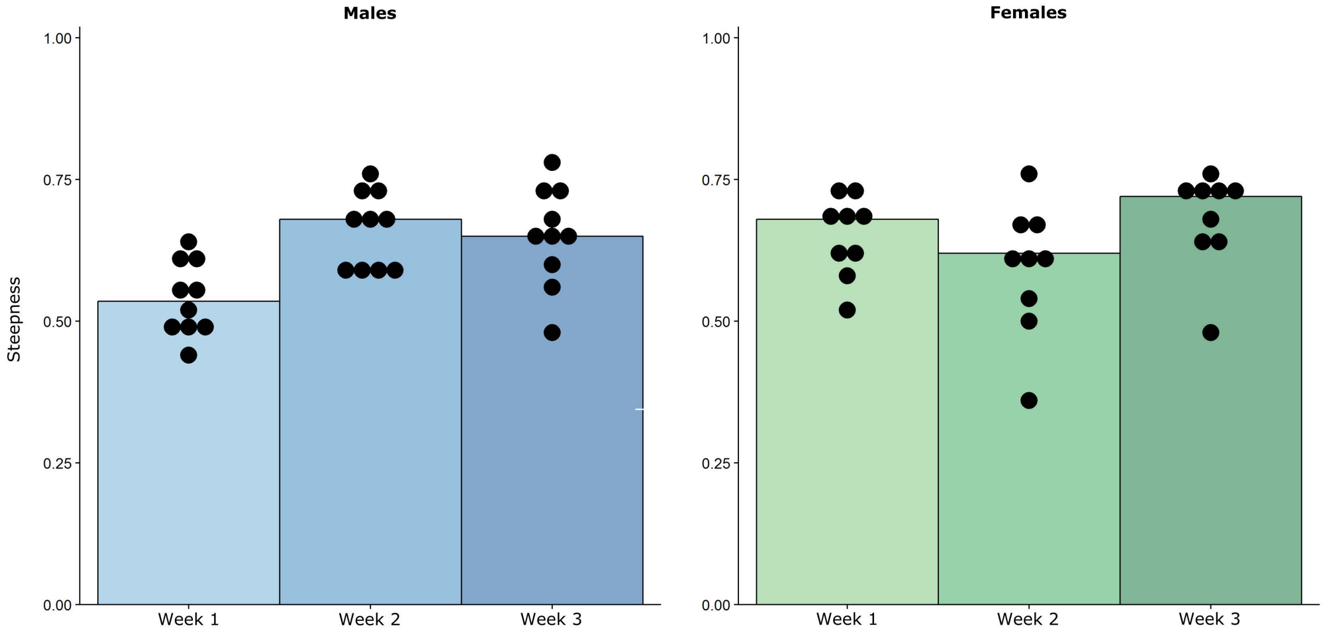

SI Fig. 2: Steepness for each cage on Week 3. Each panel represents a cage of mice and normalised DS and Ordinal rank during week 3 are plotted. A line of best fit is then plotted, representing the slope or steepness of the hierarchy considering the proportion of wins or DS. Landua's h for each cage is then presented for comparison.

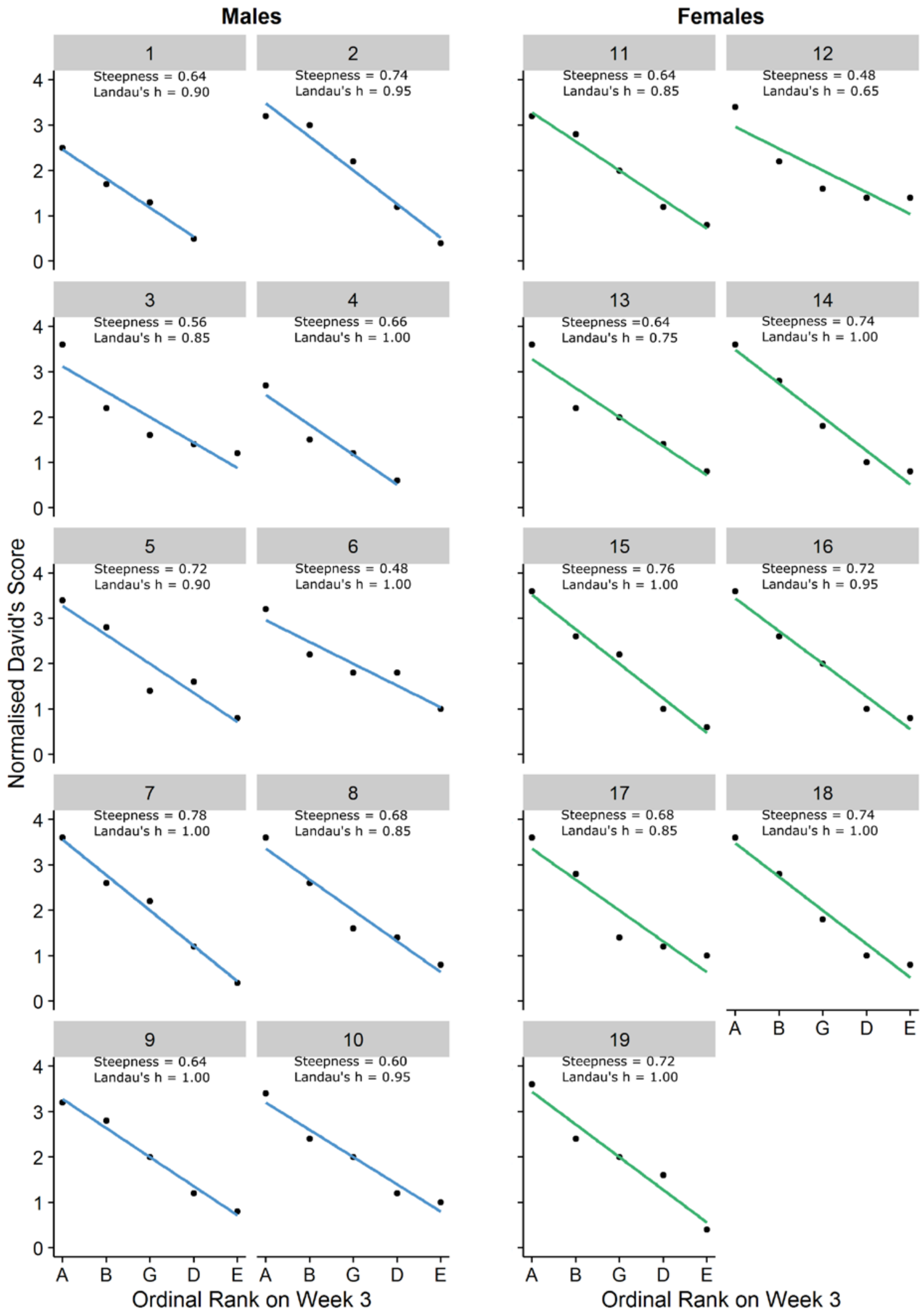

SI Fig. 3: Directional Consistency (DC) between dyads of ordinal rank at week 3. Ordinal rank of the winner at week 3 is on the y axis, and ordinal rank of loser at week 3 is on the x axis. The 3 rows for each dyad/pair represent the 3 tournaments.

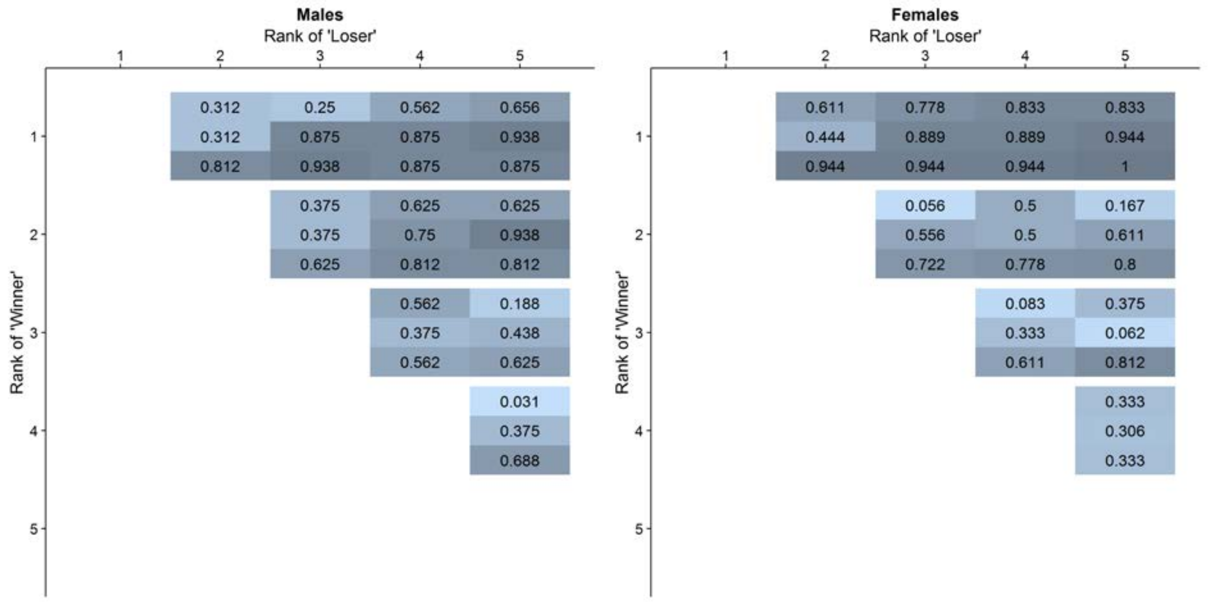

SI Fig. 4: Familiarization to familiar object over time and novel object test

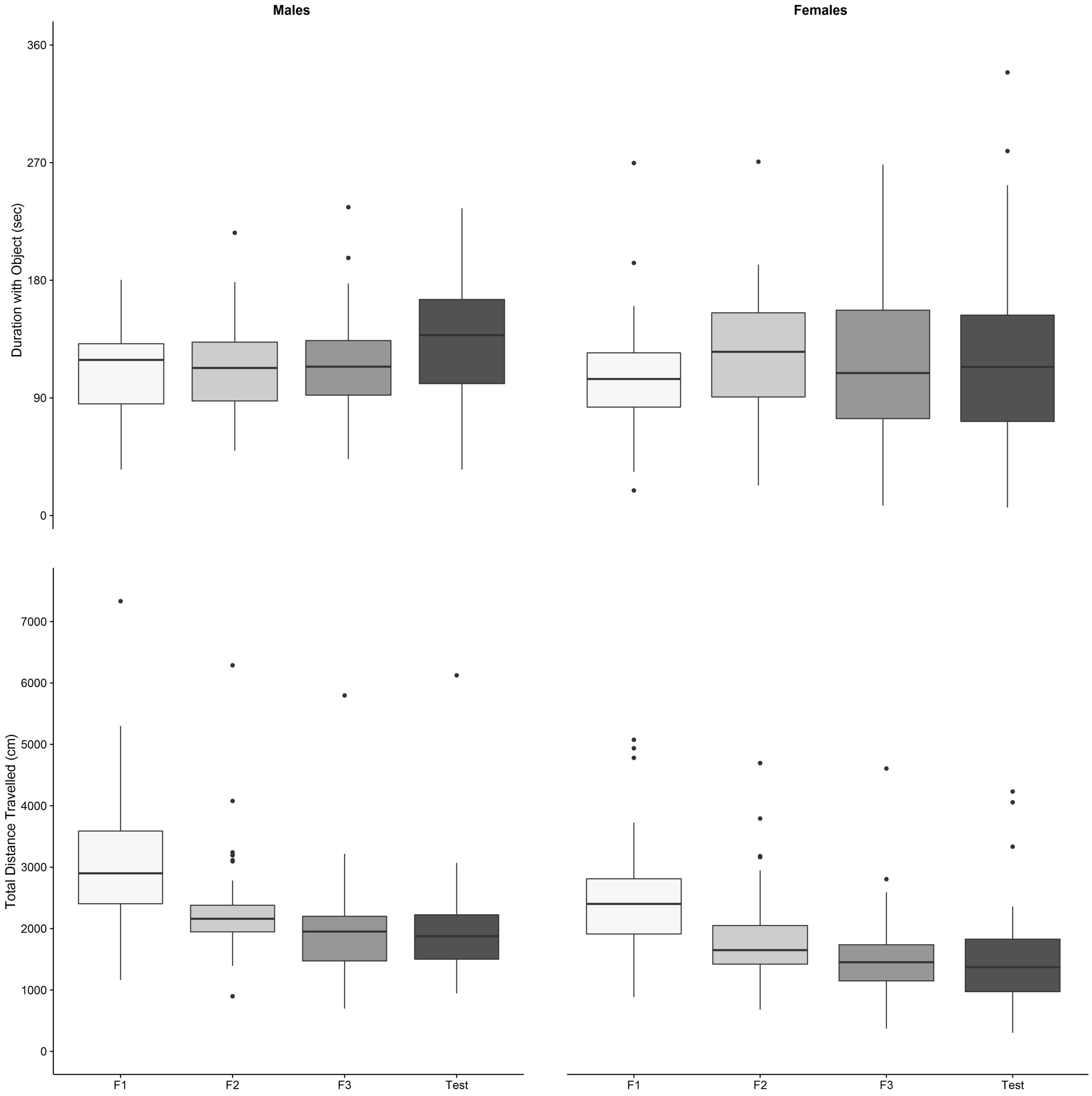

SI Fig. 5: Relationship between Novel Object Test and Cage assignment

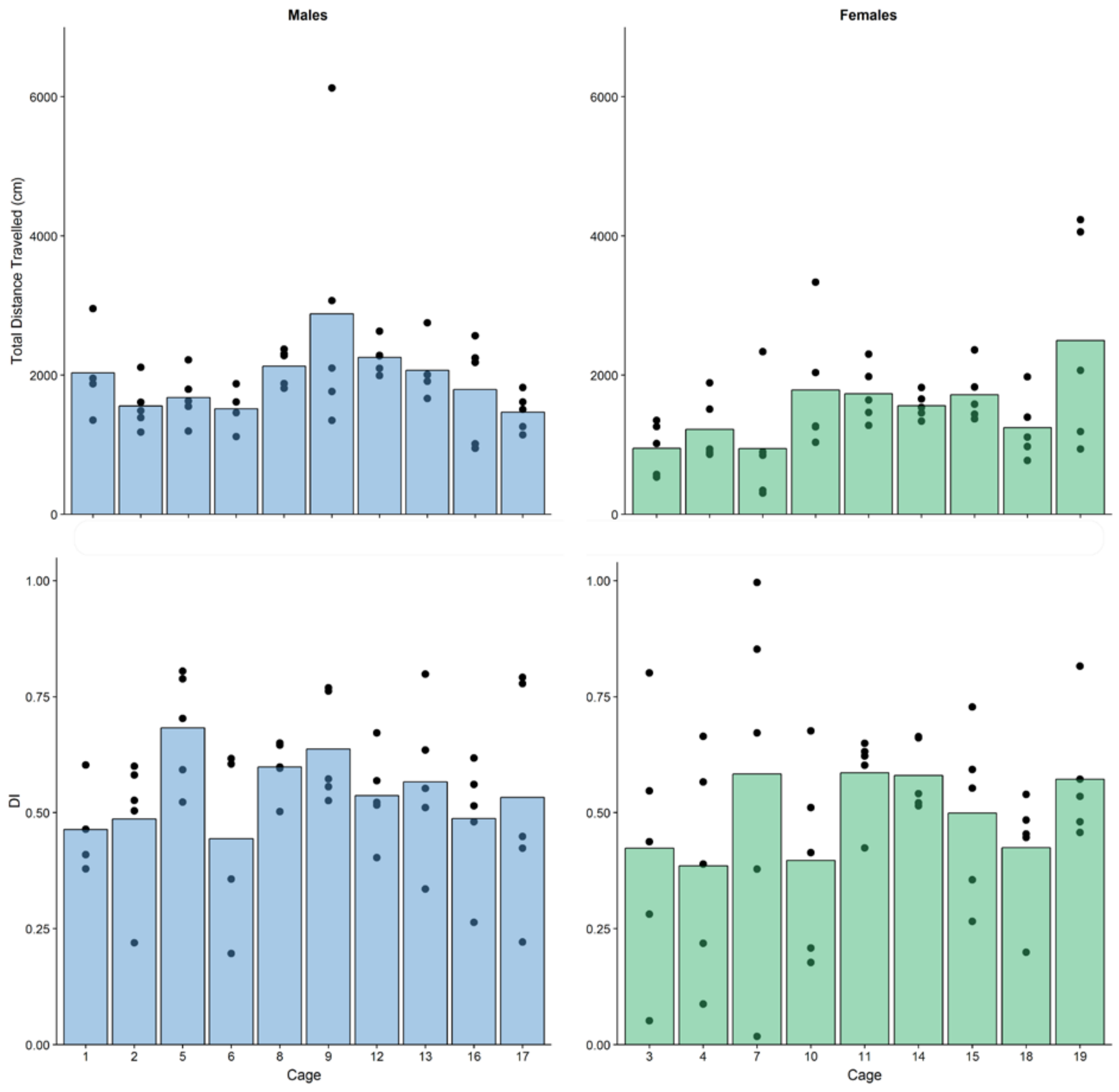

SI Fig. 6: Relationship between exploratory behaviour in the elevated zero-maze and cage assignment for males and females

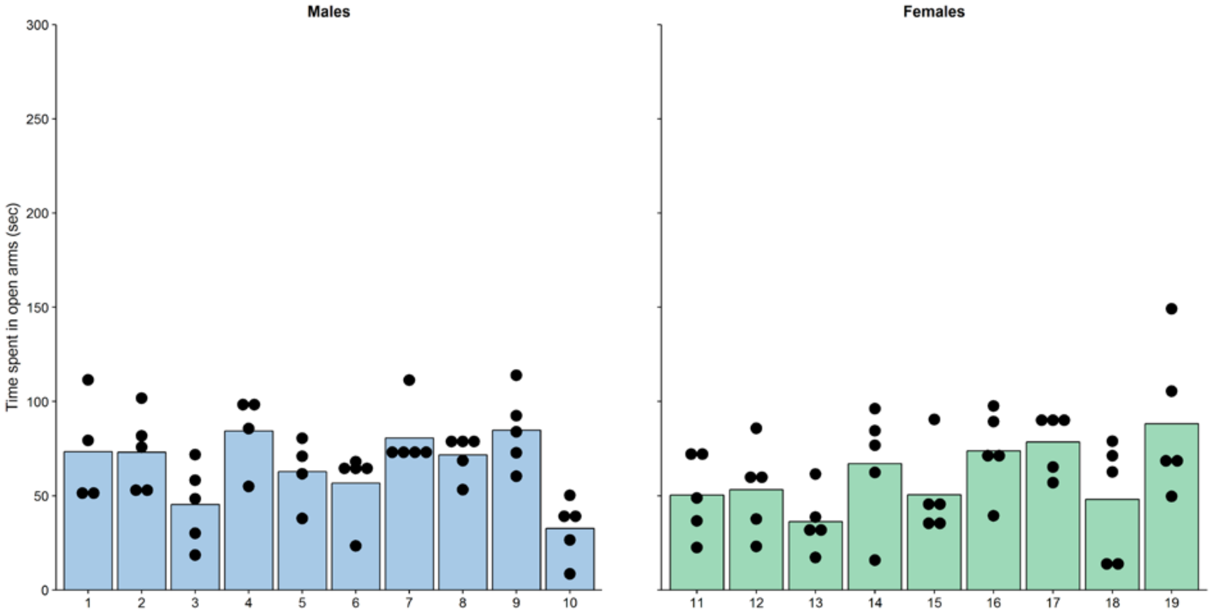

SI Fig. 7: Relationship between basal glucocorticoid levels and cage assignment for males and females

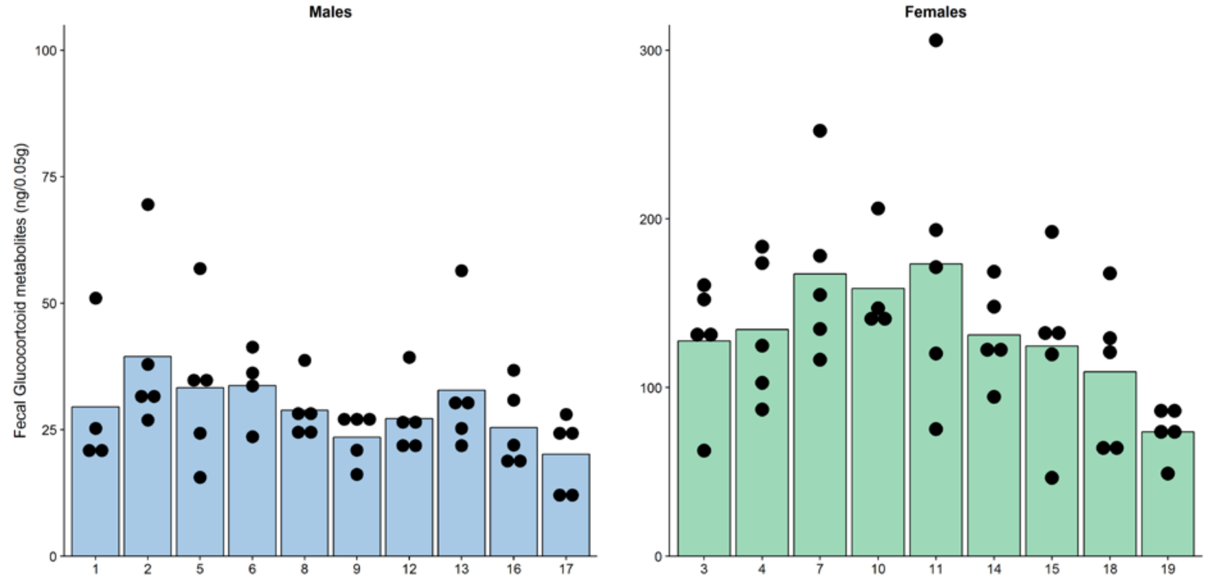

SI Fig. 8: Relationship between body mass and cage assignment for males and females

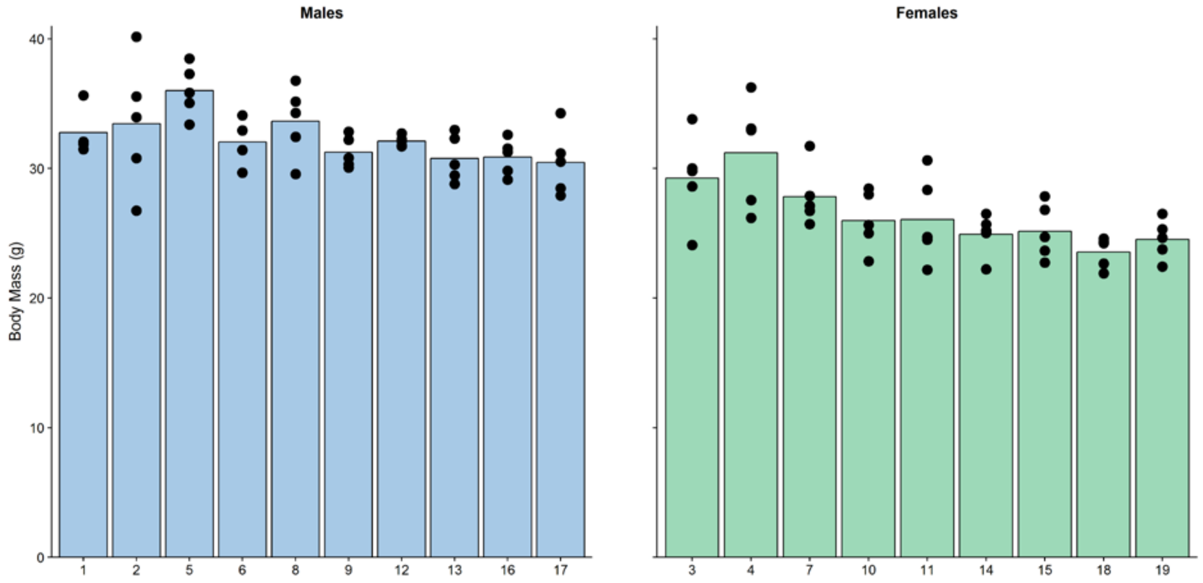

SI Fig. 9: CE apparatus

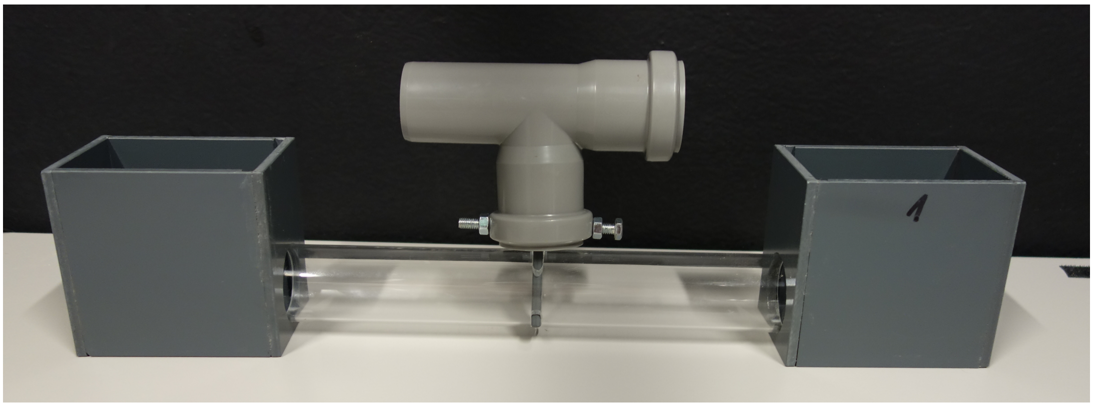

SI Fig. 10: Novel Objects

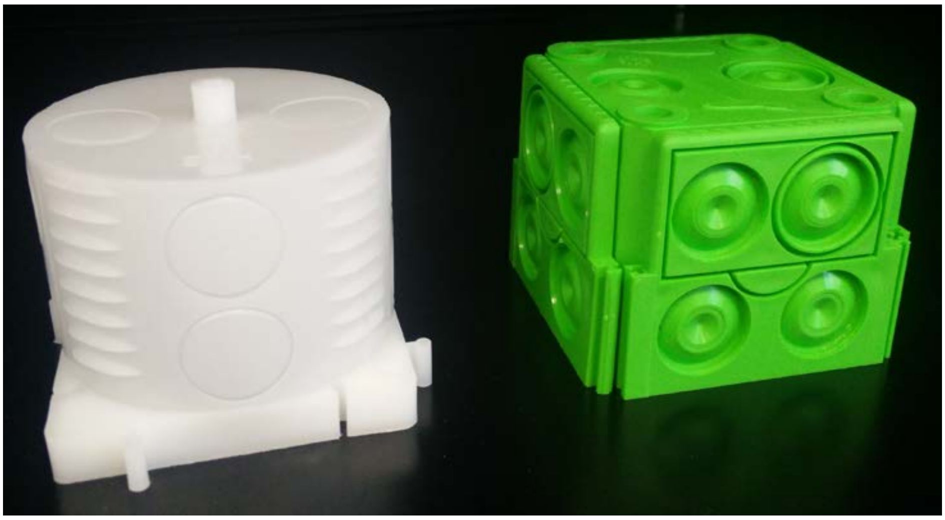

Supplement: Supplementary file 1 — Supplementary Information [file 41598_2018_24624_MOESM1_ESM.pdf]
